# How Do Polymer Coatings Affect the Growth and Bacterial Population of a Biofilm Formed by Total Human Salivary Bacteria?—A Study by 16S-RNA Sequencing

**DOI:** 10.3390/microorganisms9071427

**Published:** 2021-07-01

**Authors:** Ali Al-Ahmad, Kira Wollensak, Sibylle Rau, Diana Lorena Guevara Solarte, Stefan Paschke, Karen Lienkamp, Ori Staszewski

**Affiliations:** 1 Medical Center, Department of Operative Dentistry and Periodontology, Faculty of Medicine, University of Freiburg, Hugstetter Strasse 55, 79106 Freiburg, Germany; kirawollensak@yahoo.de (K.W.); sibylle.rau@uniklinik-freiburg.de (S.R.); diana.lorena.guevara.solarte@uniklinik-freiburg.de (D.L.G.S.); 2 Bioactive Polymer Synthesis and Surface Engineering Group, Department of Microsystems Engineering (IMTEK) and Freiburg Center for Interactive Materials and Bioinspired Technologies (FIT), University of Freiburg, Georges-Köhler-Allee 105, 79110 Freiburg, Germany; stefan.paschke@imtek.uni-freiburg.de (S.P.); karen.lienkamp@uni-saarland.de (K.L.); 3 Institut für Materialwissenschaft und Werkstoffkunde, Universität des Saarlandes, Campus, 66123 Saarbrücken, Germany; 4 Medical Center, Institute of Neuropathology, Faculty of Medicine, University of Freiburg, 79106 Freiburg, Germany; ori.staszewski@uniklinik-freiburg.de

**Keywords:** implant-associated infections, biofilm, polymer coating, 16S RNA sequencing, antimicrobial surface modification

## Abstract

Antimicrobial surface modifications are required to prevent biomaterial-associated biofilm infections, which are also a major concern for oral implants. The aim of this study was to evaluate the influence of three different coatings on the biofilm formed by human saliva. Biofilms grown from human saliva on three different bioactive poly(oxanorbornene)-based polymer coatings (the protein-repellent **PSB**: poly(oxanorbornene)-based poly(sulfobetaine), the protein-repellent and antimicrobial **PZI**: poly(carboxyzwitterion), and the mildly antimicrobial and protein-adhesive **SMAMP**: synthetic mimics of antimicrobial peptides) were analyzed and compared with the microbial composition of saliva, biofilms grown on uncoated substrates, and biofilms grown in the presence of chlorhexidine digluconate. It was found that the polymer coatings significantly reduced the amount of adherent bacteria and strongly altered the microbial composition, as analyzed by 16S RNA sequencing. This may hold relevance for maintaining oral health and the outcome of oral implants due to the existing synergism between the host and the oral microbiome. Especially the reduction of some bacterial species that are associated with poor oral health such as *Tannerella forsythia* and *Fusobacterium nucleatum* (observed for **PSB** and **SMAMP**), and *Prevotella denticola* (observed for all coatings) may positively modulate the oral biofilm, including in situ.

## 1. Introduction

Saliva and oral biofilms comprise a plethora of bacteria. A list of frequently-found genera and species in the oral cavity is included in Table 1. The most dominant groups are *Streptococcus* spp., which have been associated with dental caries [1], *Actinomyces* spp., which play a role in initial phase of caries development [2], *Fusobacterium* spp. (a bridging microorganism that can co-aggregate with early and late colonizers and hence plays a major role in the maturation of the oral biofilm), *Veillonella* spp. and *Rothia* spp. (which are considered as markers for early childhood caries) [3], *Prevotella* spp., *Tannerella* spp. and *Porphyromonas* spp. (associated with periodontitis patients) [4,5], *Neisseria* spp., and *Gemella* spp. [5], which affect the periodontal health status. While the dominant bacterial species are relatively similar among different people, the exact composition of the bacteria in saliva is individual and affected, for example, by a person’s dietary habits and biochemical parameters of the saliva such as content of lysozyme and pH value [1,6].

Thus, oral biofilms are involved in a number of dental diseases, among which caries and periodontitis are the most frequent [1,7]. A diverse array of acidogenic and aciduric species including *Streptococcus mutans*, *Lactobacilli*, *Streptococcus salivarius*, *Actinomyces* spp., *Veillonella* spp., and *Atopobium* spp. are involved in caries development [1,8]. *Tannerella forsythia*, *Prevotella denticola*, *Filifactor alocis*, *Porphyromonas gingivalis* and *Aggregatibacter actinomycetemcomitans* have been frequently associated with periodontitis [9,10]. Oral biofilms are also considered the cause of peri-implantitis, an oral implant-associated disease that is correlated with bone loss and subsequent treatment failure [11]. A shift in the subgingival oral biofilm composition with increased levels of *Fusobacterium nucleatum*, *Porphyromonas gingivalis*, *Prevotella* spp., and *Tannerella forsythia* has been shown to be associated with peri-implantitis [12,13]. From an ecological perspective, it should be emphasized that all oral infections are semi-specific and should be considered as cause of the total microbiota composition of the oral biofilm, rather than by specific species. The oral health status is the consequence of a subtle equilibrium between the host and the microbiota [14], whereby a destruction of this balance can lead to oral diseases such as caries, periodontitis, and peri-implantitis [11,15].

Oral biofilm formation is a process that is immediately initiated when a surface is in contact with human saliva. First, proteins and other organic and inorganic components of saliva adhere to the surface within seconds and form a “conditioning layer”—the so-called acquired pellicle—onto which later bacteria can settle [16]. Bacteria then use their own adhesive proteins to attach irreversibly to the surface. After an initial phase of adhesion, they start producing extracellular polymeric substances and form colonies of increasing diversity and thickness [17,18,19]. Colonies from different species form a joint extracellular matrix, which leads to the evolution of the mature biofilm [17,18,19,20]. Within the biofilm, bacteria are protected against the body’s own immune system as well as against antibiotics [21,22,23]. This is why biofilm-induced infections, for example, of oral implants, are so problematic, given that a much higher concentration of antibiotics is needed to eradicate them [22,24].

It is well known from polymer science that a number of protein-repellent polymer coatings affect the initial adhesion of bacteria on surfaces [25,26,27,28], and that various antimicrobially active polymer coatings can kill bacteria [29,30,31]. In these studies, the effect of the coating on one bacterial species is typically evaluated, while their impact on mixed bacterial populations within biofilms is much less understood. Due to the relevance of bacterial biofilms in the loss of dental implants [11,12], the aim of this study was to ascertain whether and how polymer coatings affect biofilm formation and composition after incubating these materials with total human saliva. This should answer the question of whether and how the presence of bioactive polymer coatings affect the microbial population balance of bacteria in biofilms formed by total salivary bacteria, in particular whether the known oral health-associated equilibrium within the oral biofilm would be assisted or disturbed by such materials. This knowledge would allow scientists to assess the potential of such coatings for implantology.

For this purpose, three different polymer coatings were chosen, and oral bacteria from fresh saliva were cultivated on these surfaces. After an incubation time of three days, the biofilms were harvested from the surfaces, and their most abundant constituents were analyzed by 16S-RNA sequencing.

**Table 1:** Important bacterial genera and species found in the human oral cavity, and their association with oral health or diseases.

Genus and Species	Gram Type	Growth Conditions	Clinical Relevance	References
*Actinomyces*	Gram+	facultative anaerobe	adhere to oral tissue, play an important role in biofilm formation, contribute to caries and periodontitis	[1,2,8,32]
*Actinomyces naeslundii*	Gram+	facultative anaerobe	associated with healthy microbiota, interaction with *Streptococcus mutans*, involved in initial dental biofilm formation, attaches to the pellicle	[33,34,35]
*Actinomyces radicidentis*	Gram+	facultative anaerobe	found in infected root canals of teeth and tissue	[32,36]
*Aggregatibacter actimycetemcomitans*	Gram−	facultative anaerobe	associated with peridodontitis.	[9,10]
*Brevundimonas diminuta*	Gram−	aerobe	periodontal pathogen, in subgingival niche	[37]
*Campylobacter consisus*	Gram−	anaerobe	in subgingival niche, isolated from the oral cavity of patients with gingivitis and periodontitis, but with no clear association to either disease or other human oral inflammatory diseases	[38]
*Campylobacter curvus*	Gram−	anaerobe	subgingival; no significant evidence for causing periodontal disease, but found it higher proportions at periodontitis sites as compared to healthy ones	[39]
*Campylobacter ureolyticus*	Gram−	anaerobe	gastrointestinal pathogen; present in subgingival plaque; associated with poor oral hygiene.	[40]
*Corynebacterium*	Gram+	aerobe, facultative anaerobe	found in dental plaque, associated with the formation of dental calculi	[41]
*Dialister*	Gram−	anaerobe	*D. pneumosintes* and *D. invisus* are periodontal pathogens, have been associated with refractory periodontitis, acute necrotizing ulcerative gingivitis, endodontic infections; subgingival	[37,42]
*Enterococcus*	Gram+	anaerobe	*E*. *faecalis* has been related to caries, endodontic infections, periodontitis, and peri-implantitis, biofilm former able to integrate in the oral biofilm in situ	[43]
*Filifactor alocis*	Gram+	anaerobe	associated with periodontitis.	[9,10]
*Fusobacterium nucleatum*	Gram−	anaerobe	member of the “orange complex“, periodontal pathogen, involved in primary infections of endodontal lesions, associated with subgingival plaque, peri-implantitis, gingivitis, advanced irreversible forms of periodontitis, associated with dental fluorosis, bridging microorganism which can co-aggregate with early and late colonizers, plays a major role in the maturation of the oral biofilm, one of the most abundant genera in the native biofilm	[12,13,44,45,46]
*Fusobacterium pseudoperiodonticum*	Gram−	anaerobe	isolated from periodontitis lesions and from the tongue	[47]
*Gemella sanguinis*	Gram+	facultative anaerobe	periodontal pathogen; affects the periodontal health status and is prevalent in refractory periodontitis; in subgingival niche	[5,37]
*Haemophilus parainfluenzae*	Gram−	facultative anaerobe	significantly more prevalent in periodontal health than periodontitis	[37]
*Lactobacillus*	Gram+	anaerobe	associated with carious lesions, anticaries potential, can inhibit tooth decay by limiting growth and virulence properties of *Streptococcus mutans*”, dominant genera in the pulp chamber of apical periodontitis (rat model)	[45,48,49,50]
*Lautropia mirabilis*	Gram−	facultative anaerobe	localized on the back of the tongue, significantly more prevalent in periodontal health than periodontitis	[37,51]
*Megaspahera elsdenii*	Gram−	anaerobe	one of the most abundant genera in the native biofilm	
*Megaspaera stantonii*	Gram−	anaerobe	one of the most abundant genera in the native biofilm	
*Neisseria*	Gram−	aerobic	often found resistant to antibiotics in periodontal patients undergoing therapy; affect the oral health status;	[5,52]
*Neisseria mucosa*	Gram−	aerobic	Found in gingival plaque, affect the periodontal health status	[53]
*Neisseria subflava*	Gram−	aerobe	populates the tongue dorsum, affects the periodontal health status	[53]
*Olsenella*	Gram+	obligate anaerobe	associated with carious lesions	[49]
*Porphyromonas gingivalis*	Gram−	anaerobe	member of the “red complex”, involved in periodontitis, primary infections of endodontal lesions, associated with peri-implantitis	[34,54]
*Prevotella denticola*	Gram−	anaerobe	dominant bacteria in oral lichen planus; abundance in the oral cavity can lead to mucosal inflammation and oral lichen planus; associated with periodontitis.	[4,5,9,10,12,13,55]
*Prevotella melaninogenica*	Gram−	anaerobe	commensal bacterium, member of normal human oral microbiota	[56]
*Rothia mucilaginosa*	Gram+	aerobe	found on the tongue (up to 6%), found at high abundance on oral leukoplakia, significantly more prevalent in periodontal health than periodontitis, marker for early childhood caries	[3,37,57]
*Schaalia odontolytica*	Gram+	anaerobe	associated with healthy microbiota	[46]
*Selenomonas* sp. oral taxon 136	Gram−	anaerobe	periodontal pathogen, significantly prevalent in refractory periodontitis, in subgingival niche	[37]
*Staphylococcus schleiferi*	Gram+	facultative anaerobe	veterinary pathogen, but can also cause opportunistic infections in humans	[58]
*Streptococcus koreensis*	Gram+	facultative anaerobe	associated with subgingival dental plaque/periododitis lesion	[58]
*Streptococcus parasanguinis*	Gram+	facultative anaerobe	“pioneer species”, colonizes teeth fast; involved in the periodontal biofilm, periodontal pathogen; dynamic interaction with *A. actinomycetemcomitans*, a late colonizer and periodontal pathogen; together with *A. actinomcetemcomitans* and *Filifactor alocis*: may be biomarker for periodontitis	[40,45,59]
*Streptococcus pneumoniae*	Gram+	facultative anaerobe	associated with periodontal health; associated with sinusitis	[60]
*Streptococcus salivarius*	Gram+	facultative anaerobe	colonizes the tongue associated with carious lesions	[45,49]
*Streptococcus sanguinis*	Gram+	facultative anaerobe	“pioneer species”, colonizes teeth fast; important for supragingival biofilm formation; may suppress the generation of caries; antagonism between *S. sanguinis* and *S. mutans*; associated with oral health	[37,61]
*Streptococcus thermophilus*	Gram+	facultative anaerobe	poor biofilm former	[32]
*Tannerella forsythia*	Gram−	anaerobe	member of the “red complex”; in subgingival niche; associated with periodontal disease and peri-implantitis	[4,5,9,10,12,13,62]
*Tannerella sp. Oral taxon HOT-286*	Gram−	anaerobe	associated with periodontal health; in subgingival niche; may provide protection from acquisition of *T. forsythia*	[62]
*Veillonella*	Gram−	anaerobe	found on the tongue, the buccal mucosa, and in saliva, interactions between *Streptococcus* species and *Veillonella* species in the early stages of oral biofilm formation; associated with caries, consistently resistant to antibiotics in periodontal patients undergoing therapy; presence in children may predict the development of future caries, indication for poor oral health.	[1,3,8,52]
*Veillonella atypica*	Gram−	anaerobe	early colonizers in oral biofilm formation; periodontal pathogen significantly prevalent in refractory periodontitis; subgingival	[37,52]
*Veillonella dispar*	Gram−	anaerobe	early colonizers in oral biofilm formation	[52]
*Veillonella parvula*	Gram−	anaerobe	associated with severe early childhood caries and intraradicular infections (abscess, apical root canals, and dentinal tubules); early colonizers in oral biofilm formation	[52]

## 2. Materials and Methods

### 2.1. Coating Preparation

**Figure 1:**
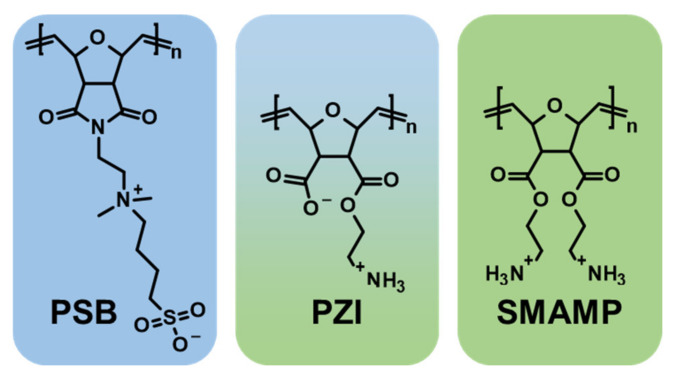
The effect of polymer coatings made from the protein-repellent PSB, the protein-repellent and antimicrobial PZI, and the mildly antimicrobial SMAMP on the growth and microbial composition of oral biofilms was studied.

The sample substrates had a size of 15 × 15 mm. The polymer coatings were fabricated as previously reported [63,64,65], and as described in the experimental section. In short, the sample substrates (silicon wafer pieces with a size of 15 × 15 mm that had been pre-treated with the cross-linker benzophenone-3-(ethoxysilane)) [65] were spin-coated with solutions containing the different polymers and the cross-linker pentaerythritol-tetrakis-(3-mercaptopropionat). The polymer coatings obtained were UV irradiated. This caused covalent attachment of the polymers to the substrate and simultaneous formation of a polymer network. The surface-attached polymer networks were then washed with different solvents to extract unbound polymer chains, and dried. The polymer coatings obtained (Figure 1) had an average thickness of 190 nm (**PSB**), 155 nm (**PZI**), and 180 nm (**SMAMP**), as determined by ellipsometry. The **PSB** samples obtained were stored until use, and **SMAMP** and **PZI** were treated with HCl to remove the N-boc protective groups, rinsed and dried directly before use.

**PSB** samples: First, a solution of pentaerythritol-tetrakis-(3-mercapto-propionat) (0.1 mL) in trifluoroethanol (TFE, 5 mL) was prepared (solution A). **PSB** (30 mg) was dissolved in solution A (0.25 mL), and TFE (0.75 mL) was added. The polymer was spin-cast onto pre-treated silicon wafers using the following parameters: 3000 rpm, 500 rpm s^−1^, 20 s. All wafers were irradiated with UV-light at a wavelength of 254 nm at 3 J cm^−2^ and extracted in TFE afterwards. The wafers were then rinsed with ethanol and dried in a stream of nitrogen.

**SMAMP** samples: First, a solution of pentaerythritol-tetrakis-(3-mercapto-propionat) (0.1 mL) in dichloromethane (DCM, 5 mL) was prepared (solution B). **SMAMP** (20 mg) was dissolved in solution B (0.25 mL) and chloroform (0.6 mL) was added. The polymer was spin-cast onto pre-treated silicon wafers using the following parameters: 3000 rpm, 1000 rpm s^−1^, 10 s. All wafers were irradiated with UV-light at a wavelength of 254 nm at 3 J cm^−2^ and extracted in DCM afterwards. The wafers were then rinsed with ethanol and dried in a stream of nitrogen. At 24 h before intended use, the samples were immersed into HCl (4 M in dioxane) over night. Afterwards, they were then rinsed with ethanol and dried in a stream of nitrogen.

**PZI** samples: **PZI** (20 mg) was dissolved in water (0.1 mL) and methanol (0.9 mL). The polymer was spin-cast onto pre-treated silicon wafers using the following parameters: 3000 rpm, 1000 rpm s^−1^, 30 s. All wafers were irradiated with UV-light at a wavelength of 254 nm at 0.3 J cm^−2^. The wafers were dipped into glacial acetic acid and dried in a stream of nitrogen.

### 2.2. Biofilm Growth

Biofilm growth on the different materials was conducted as previously described [63], with slight modifications. The negative controls were uncoated silicon wafer pieces and the positive control was an uncoated silicon wafer piece treated with 0.2% chlorhexidine digluconate (**CHX**) solution for 24 h. All samples were placed in a twelve-well cell culture plate (Greiner Bio-One, Frickenhausen, Germany) using sterilized tweezers. Stimulated human saliva from three healthy volunteers was taken after chewing paraffin tablets. All salivary samples were used freshly to avoid any additional modifications of the microbial composition of the inoculum. All saliva samples were vortexed for 30 sec, pooled and stored in 50 mL Falcon tubes (Greiner Bio-One, Frickenhausen, Germany). The exclusion criteria for the volunteers were severe systemic diseases, the use of antibiotics and antimicrobial mouthwashes within the last three months, pregnancy, and lactation. All volunteers gave their written consent. The study was approved by the ethics committee of the Albert-Ludwigs-University Freiburg (381/15 and 91/15).

Each sample was inoculated with 1000 µL of the pooled saliva. For initial microbial adhesion, all samples were incubated at 37 °C, 95% humidity, and 5% CO_2_ for 2 h. After this initial phase of biofilm formation, 500 µL of tryptic soy broth (TSB, Merck, Darmstadt, Germany) was added to each sample, followed by an incubation for 24 h. After this second incubation time, 750 µL of the culture in each well was discarded and fresh TSB were added to further incubate the sample for additional 24 h, after which the culture medium was refreshed again. The samples were incubated under the same conditions for 24 h. The biofilm formation took 72 h overall. Biofilm coated samples were each washed with 1000 µL reduced transfer fluid (RTF) [66] to remove non-adherent bacteria. The biofilm was then carefully scraped from the surface and stored in RTF at − 80 °C until it was used for 16S-RNA sequencing. The microbial composition of total salivary bacteria, and that of the native biofilm formed on the uncoated silicon wafers, was also analyzed.

### 2.3. RNA Isolation

Biofilm samples were mixed 2:1 with lysis buffer comprising 5 mg/mL lysozyme (L6876 Sigma-Aldrich, Munich, Germany), 10 mg/mL proteinase K (3115836001, Roche Diagnostics, Mannheim, Germany), 9500 U/mL PNGase F (P0704, New England Biolabs, Frankfurt, Germany), 3000 U/mL RNAse Inhibitor (M0314, New England Biolabs, Frankfurt, Germany), 0.5% NP40 (ab142227, Abcam, Cambridge, UK), 5 mM Tris-HCl and 0.05 mM EDTA (TE pH 8 – 12090015, Thermo Fisher Scientific, Braunschweig, Germany). The total biofilm formed on each sample surface was used for the analysis. Quantification was performed by measuring the extractable total RNA, where 100 µL lysis buffer was used to extract the total RNA. Samples were then incubated for 30 min at 37 °C, followed by a 30-min incubation at 55 °C. Immediately afterwards, total RNA was extracted using the Picopure RNA extraction kit (KIT0204, Thermo Fisher Scientific, Braunschweig, Germany) following the manufacturer’s recommendations, including a DNAse incubation step as per the manufacturer’s recommendations. The total RNA was eluted in 14 µL H_2_O, and the RNA concentration was measured with a Qubit high sensitivity RNA kit (Q32852, Thermo Fisher Scientific, Braunschweig, Germany). Lysates were treated with DNAse and DNA content was measured with a Qubit ds High Sensitivity Assay kit in selected samples. In both, RNA and cDNA samples, no dsDNA was detectable.

### 2.4. cDNA Synthesis and 16S Transcript Amplification

cDNA was synthesized using the Superscript III kit (18080051, Thermo Fisher Scientific, Braunschweig, Germany) with 2 µM 16S gene-specific primer 1492R as per the manufacturer’s recommendations [67]. For this, 8 µL of isolated RNA was added to 1 µL of gene specific primer and 1 µL of 10 mM dNTP mix. This mix was incubated at 65 °C for 5 min before adding 10 µL of cDNA Synthesis kit (2 µL 10× RT buffer, 4 µL 25 mM MgCl2, 2 µL 0.1 M DTT, 1 µL 40U/µL RNAseOut and 1 µL 200U/µL SuperScript III RT) and incubating for 50 min at 50 °C followed by 5 min at 85 °C.

A total of 10 µL of cDNA was then further amplified using 1.25 µL of 10 µM each of the same gene-specific primers 27F and 1492R with 12.5µL of Q5 High fidelity PCR master mix (M0492, New England Biolabs, Frankfurt, Germany). PCR was performed for 30 s at 98 °C followed by 35 cycles of 10 s at 98 °C, 30 s at 63 °C, and 30 s at 72 °C. A final extension of 2 min at 72 °C was performed. The PCR product was purified by means of a 1:1 incubation with Ampure XP beads (A63881, Beckman Coulter Biomedical, Munich, Germany) as per the manufacturer’s instructions. Two no RNA samples were run in parallel for cDNA synthesis and PCR and yielded no detectable PCR product as a control for potential contamination during sample preparation.

### 2.5. Sequencing Library Preparation and MinION Sequencing

Multiplexed sequencing libraries were prepared using the Ligation Sequencing Kit (SQK-LSK109, Oxford Nanopore Technologies (ONT), Oxford, UK) in conjunction with the native barcoding expansion kit ((EXP-NBD104, ONT), Oxford, UK) as per protocols provided by the manufacturer. In short, 50–60 fmol of PCR product were end repaired and dA-tailed using the NEBNext Ultra II End repair / dA-tailing Module (E7546, New England Biolabs [NEB], Frankfurt, Germany) by incubating 24 µL of PCR product in water with 1.75 µL Ultra II End-prep reaction buffer and 1.5 µL Ultra II End-prep enzyme mix at 20 °C for 10 min followed by incubation at 65 °C for 5 min. End repaired PCR product was isolated using 30 µL Ampure XP beads (A63881, Beckman Coulter, Krefeld, Germany) and resuspended in 11.5 µL water.

A total of 11.25 µL of end-repaired purified PCR product was then barcoded by incubation with 1.25 µL of native barcode and 12.5 µL of NEB Blunt/TA Ligase Master Mix (M0367, NEB) for 10 min at room temperature. Barcoded samples were subsequently purified with 25 µL Ampure XP beads and resuspended in 12 µL water. The barcoded sample concentration was measured using the Qubit dsDNA HS kit (Q32851, Thermo Fisher Scientific, Braunschweig, Germany).

Equimolar amounts of barcoded samples were pooled to yield 100–120 fmol of pooled barcoded samples in a total volume of 65 µL. Each pool was then incubated with 5 µL Adapter Mix II, 20 µL NEBNext Quick Ligation Reaction Buffer (B6058, NEB) and 10 µL Quick T4 DNA Ligase (E6057, NEB) for 10 min at room temperature. Adapter ligated sample pools were purified using 50 µL of Ampure XP beads and two washes with 250 µL of Short Fragment Buffer prior to being resuspended in 15 µL of Elution Buffer.

Sample pools were quantified using the Qubit dsDNA HS kit and stored at 4 °C until sequencing was performed. For each sequencing run, 10 to 15 fmol of sequencing library were used, with flow cell preparation and sample loading performed according to manufacturer’s protocol.

In total, four to five replicates of uncoated PSB, SMAMP, and PZI coated surfaces were sequenced alongside one CHX treated uncoated sample and two sputum samples to test for initial bacterial content prior to biofilm growth. For each sequencing library, twelve biofilm samples were combined in a randomized fashion to reduce batch effects due to library preparation and sequencing-induced technical variation. Sequencing libraries were sequenced on a MinION R9 flow cell for 18–24 h following the manufacturer’s recommendations.

### 2.6. Data Analysis

Fast5 files obtained from the sequencing runs were base-called and demultiplexed using a high accuracy base-calling mode with standard settings with the GPU-based guppy software (ONT, Oxford, UK) version 4.2.2). Base-called fastq files were then further processed to identify bacterial populations using the centrifuge software version 1.0.4beta in conjunction with a precompiled index for ‘bacteria, archea, viruses, human’ version 12/06/2016 as provided by the centrifuge package providers [68]. Visualization and data analysis of the centrifuge output was performed using basic R functions [69] as well as R packages ggplot2 and pavian (R package version 1.2.0, https://github.com/fbreitwieser/pavian, accessed on 11 March 2021) [70,71]. Relative abundance was calculated on the basis of the total amount of sequences obtained per sample. Species or genera constituting less than 1% in any sample were excluded from analysis.

Alpha and beta diversity measures were calculated using the R package vegan (Community Ecology Package, R package version 2.5-7., https://CRAN.R-project.org/package=vegan, published by J. Oksanen, F. Guillaume Blanchet, Michael Friendly, Roeland Kindt, Pierre Legendre, Dan McGlinn, Peter R. Minchin, R. B. O’Hara, Gavin L. Simpson, Peter Solymos, M. Henry H. Stevens, Eduard Szoecs, and Helene Wagner 2020, accessed on 11 March 2021) and visualized using the ggplot and pheatmap packages (Raivo Kolde 2019: R package version 1.0.12. https://CRAN.R-project.org/package=pheatmap, access on 11 March 2021).

## 3. Results

Three poly(oxanorbornene)-based polymer coatings with different bioactivity profiles were chosen for this study to evaluate their effects on the microbial composition of biofilms formed by total human salivary bacteria. The poly(oxanorbornene)-based poly(sulfobetaine) (PSB) is protein-repellent but not antimicrobially active [63,64,72]. The polycationic (SMAMP) is intrinsically active against bacteria, albeit only mildly, with about 90% growth reduction of Escherichia coli and *Staphylococcus aureus* bacteria after two hours of incubation [65]. The third coating is a poly(carboxyzwitterion) (PZI) with charge-switchable properties [63,64]. It is protein-repellent under physiological conditions, but becomes intrinsically antimicrobial in the presence of bacteria, presumably because they secrete acidic metabolites [63,64]. The polymer coatings obtained were very smooth and fully covered the substrate, as evaluated by atomic force microscopy. Due to the low thickness of the coating, which was below 200 nm in all cases, the macroscopic mechanical properties of the substrate were not altered. All coatings were non-toxic to human keratinocytes, as determined by the AlamerBlue assay. Full details of the physical-chemical characteristics and the bioactivity of these coatings, including their compatibility with human cells, can be found in the references given above. To assess the effect of these different bioactivity profiles on biofilm microbiota, they were incubated with unstimulated human saliva for biofilm film formation by the salivary bacteria. After 72 h, the biofilms were harvested and analyzed by means of 16S RNA sequencing.

The biofilm formed on the different samples was exemplarily verified by confocal laser scanning microscopy and live/dead staining. Example images have now been added in the Supplementary Materials (Figure S1).

### 3.1. Total Amount of Biofilm RNA

**Figure 2:**
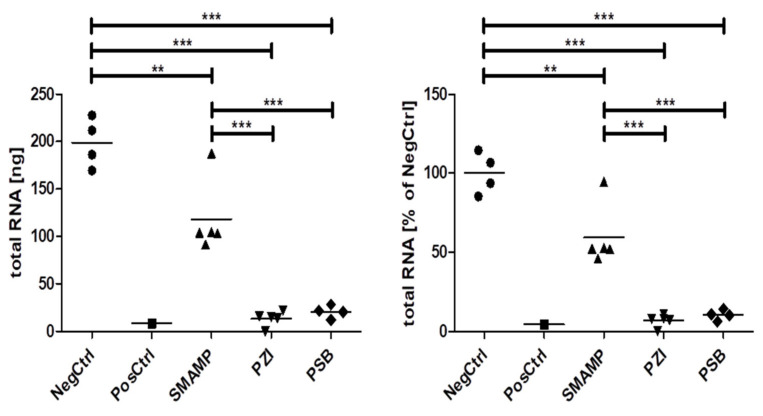
Total extractable RNA in the harvested biofilms as a percentage of the mean of the negative control (**NegCtrl =** uncoated silicon wafer, **PosCtrl** = uncoated silicon wafer with additional CHX). Stars depict significant differences as determined by one-way ANOVA with Tukey’s post-hoc test. ** *p* < 0.01, *** *p* < 0.001.

The total amount of extracted RNA was also determined, as a measure of the amount of living bacteria in the biofilm. As shown in Figure 2, this amount significantly varied by surface coating type (see also Table S1 in the supporting information). **SMAMP** coatings reduced the amount of extractable RNA by about 40% when compared to uncoated surfaces (NegCtrl in Figure 2); **PZI** and **PSB**-coated surfaces even had 90% less extractable RNA (Figure 2). No significant differences could be seen between **PZI** and **PSB**-coated surfaces regarding total extractable RNA.

### 3.2. Microbial Composition of Human Saliva and Biofilm Composition on All Surfaces

**Figure 3:**
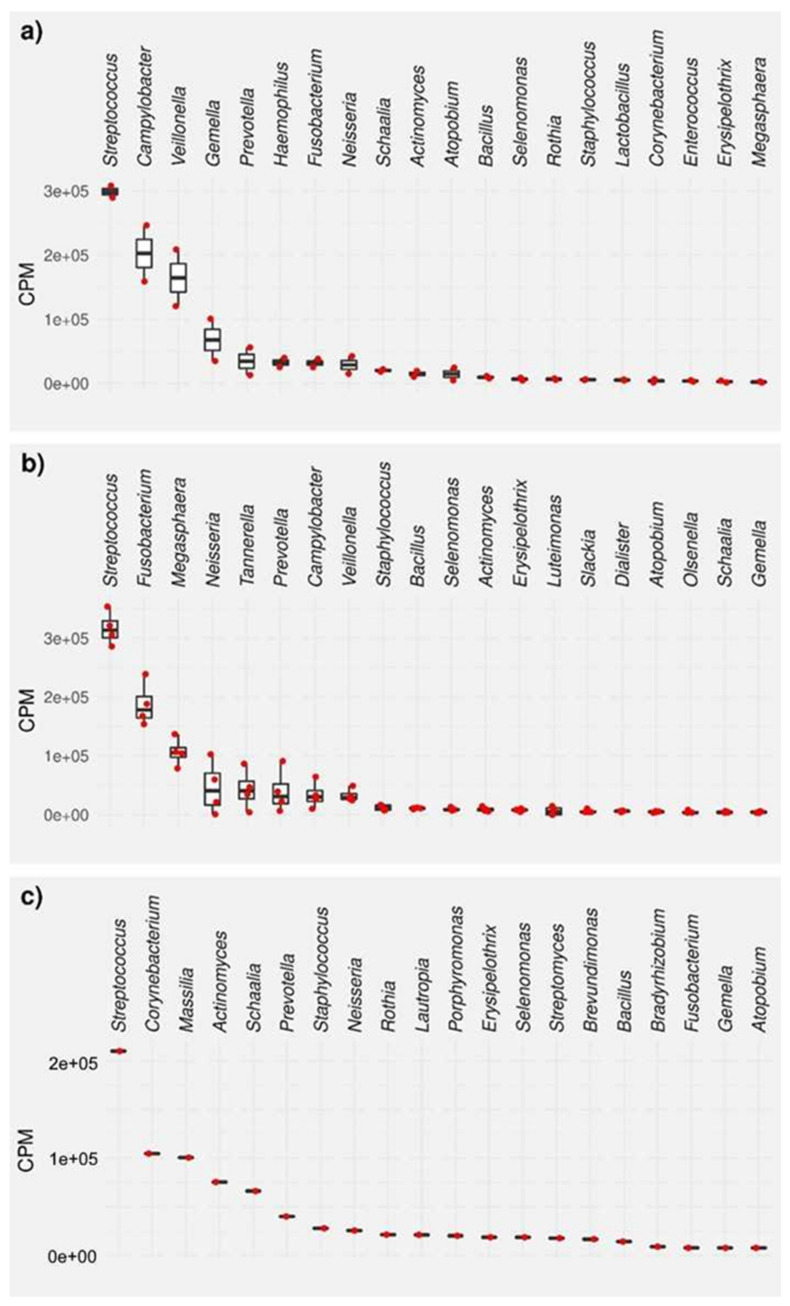
Top 20 most abundant bacterial genera, presented as CPM, for (**a**) bacteria found in saliva, (**b**) bacteria harvested from native biofilms grown on uncoated substrates, and (**c**) bacteria harvested from biofilms grown on uncoated substrates to which the disinfectant chlorhexidine digluconate (**CHX**) had been added.

**Figure 4:**
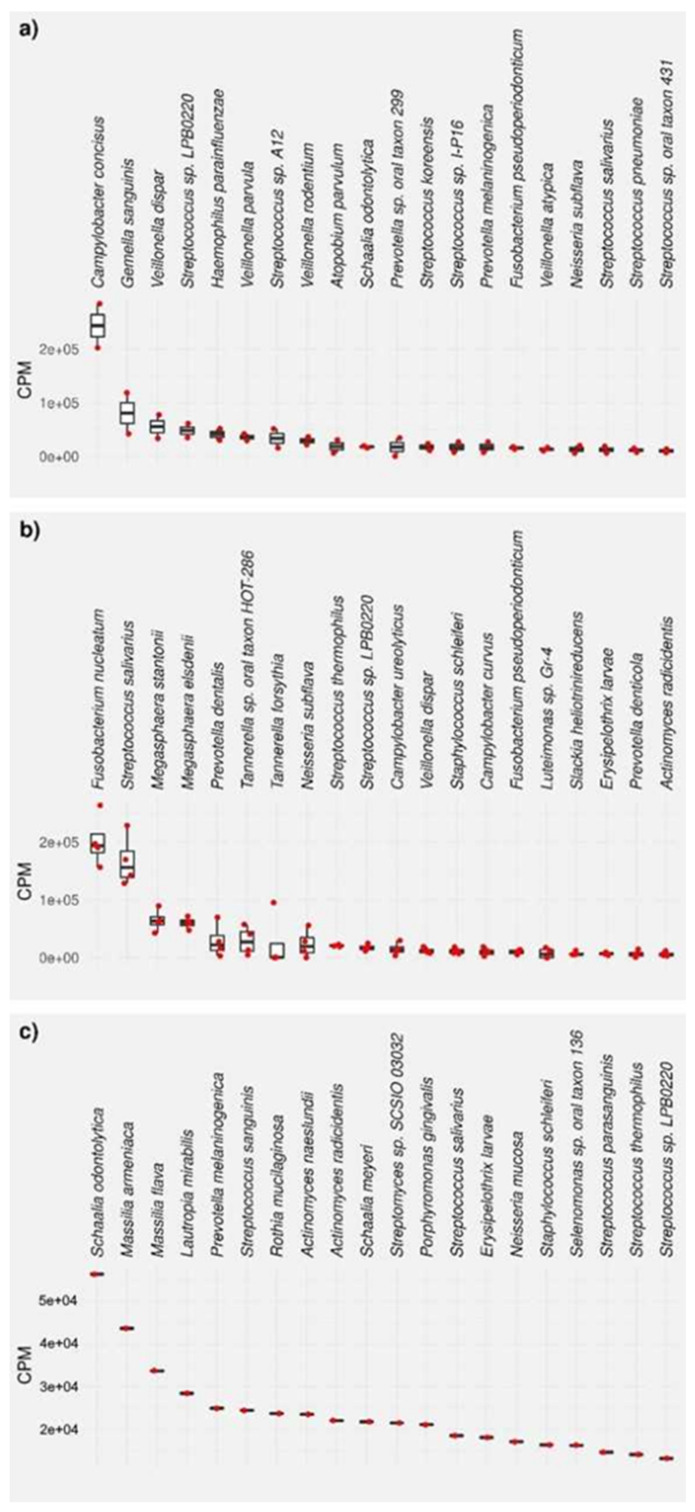
Top 20 most abundant bacterial species, presented as CPM, for (**a**) bacteria found in saliva, (**b**) bacteria harvested from native biofilms grown on uncoated substrates, and (**c**) bacteria harvested from biofilms grown on uncoated substrates to which the disinfectant chlorhexidine digluconate (CHX) had been added.

**Figure 5:**
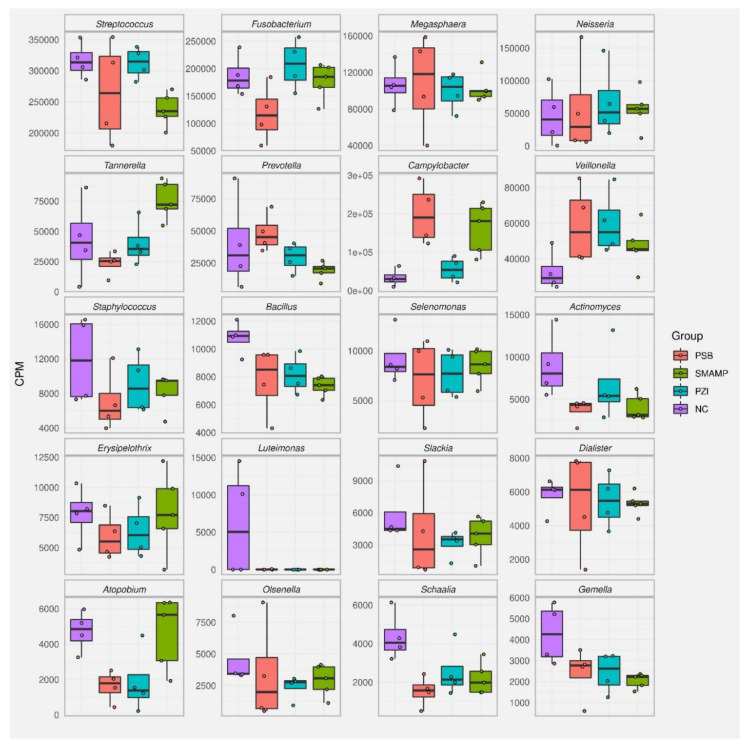
Top 20 most abundant bacterial genera, presented as CPM, for bacteria harvested from native biofilms grown on uncoated substrates (**NC**, purple), and bacteria harvested from biofilms grown on polymer-coated substrates (**PSB**, red; **PZI**, turquois; **SMAMP**, green).

**Figure 6:**
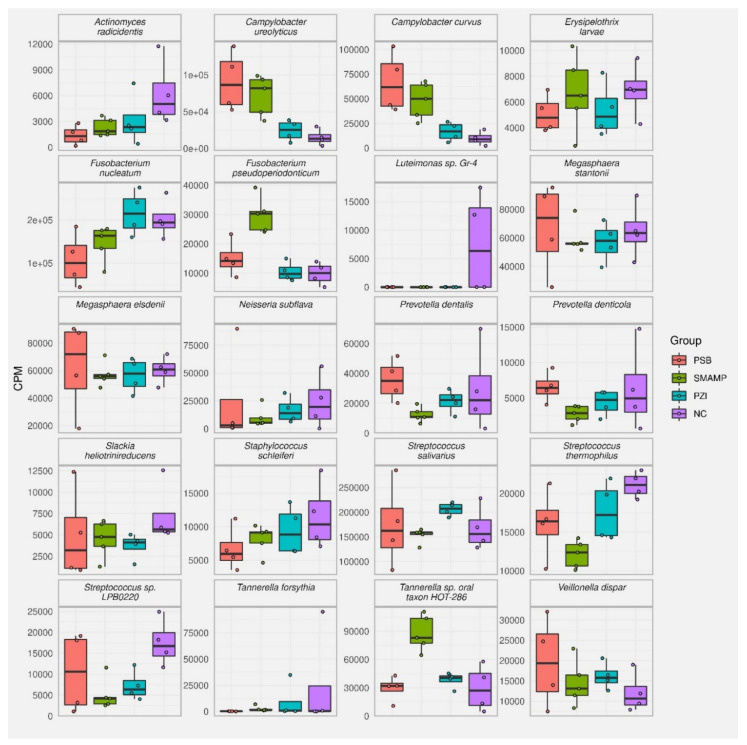
Top 20 most abundant bacterial species, presented as CPM, for bacteria harvested from native biofilms grown on uncoated substrates (**NC**, purple), and bacteria harvested from biofilms grown on polymer-coated substrates (**PSB**, red; **PZI**, turquois; **SMAMP**, green).

The bacterial composition of the biofilms was then analyzed for each biofilm sample, except for one PZI sample, which yielded no detectable RNA after isolation, by sequencing of the 16S ribosomal RNA molecule which allows identifying individual bacterial species within a complex mixture. The results are reported as the top 20 species and genera for each sample type in counts per million (CPM) and percent (Figure 3, Figure 4, Figure 5 and Figure 6, and Tables S2–S5 in the Supporting Information). Due to the number of processing steps required, and the method accuracy, bacteria present in quantities below 1% relative abundance were not considered for further analysis. The 20 most abundant bacterial genera thus identified are presented in Figure 3 and the 20 most abundant bacterial species identified in these samples are presented in Figure 4 for (a) bacteria found in saliva, (b) bacteria harvested from biofilms grown on uncoated substrates (called native biofilms in the following), and (c) bacteria harvested from biofilms grown on uncoated substrates to which the disinfectant chlorhexidine digluconate (CHX) had been added. These numbers have also been converted into their relative abundance in the biofilm (in percent, Tables S2–S5). It should be kept in mind that the amount of 16S rRNA also correlates to a high number of ribosomes, which in turn correlates with the activity of bacteria, since active bacterial cells contain a higher number of ribosomes than inactive ones.

In both saliva and the native biofilm, *Streptococci* were the most abundant bacteria, with a relative abundance of 31.2% and 35.6%, respectively, in line with their importance in oral biofilm formation. Four of the five following most abundant genera in saliva (*Campylobacter*, *Veillonella*, *Gemella*, and *Haemophilus*) had a drastically diminished abundance in the native biofilm compared to saliva. The counts of *Campylobacter* spp. and *Veillonella* spp. were reduced by one order of magnitude (from 31.2% to 3.8%, and from 17.2% to 3.7%, respectively), while *Gemella* spp. (from 7.1% to 0.5%) and *Haemophilus* spp. (from 3.4% to <0.5%) became insignificant in the formed biofilm. The relative abundance of *Prevotella* spp.—the fifth most abundant genus in saliva—and *Neisseria* spp. (ranked seventh) was more or less unchanged in the native biofilm (from 3.6% to 4.5%, and from 3.0% to 5.2%, respectively). Interestingly, *Fusobacterium* spp.—which only had an abundance of 3.3% in saliva—had a share of 21.0% in the native biofilm, thus taking the second rank after *Streptococcus* spp. Moreover, genera like *Megasphaera* and *Tannerella*, which held little to no importance in the saliva sample, increased their relative abundance from 0.2% to 12%, and from < 0.2% to 4.8% in native biofilm. The slightly higher relative abundance of *Staphylococcus* spp. and *Bacillus* spp. in the biofilm compared to their abundance in saliva (from 0.6% to 1.3%, and from 1.0% to 1.2%) might hold relevance due to their pathogenic potential.

The analysis of alpha-diversity (Figure S2) showed a significant increase of species richness (increase of alpha-diversity) on samples coated with SMAMP as compared with the negative control (uncoated samples) (*p* = 0.013). The species richness also increased significantly on the SMAMP surface in comparison to the samples coated with PZI (*p* = 0.017). The beta-diversity (Figure S3) was analyzed in order assess differences between the microbial communities on the differently coated surfaces and the negative control. The beta-diversity revealed significant differences between the SMAMP surface and the negative control (*p* = 0.014). Significant differences of the microbial composition were also revealed by the beta-diversity between and the PZI and SMAMP surface (*p* = 0.01).

In the biofilm grown in the presence of **CHX**, *Streptococcus* was still the most abundant genus (with 25.2%), and other genera that were insignificant in both saliva and the native biofilm had a large share in the **CHX**-treated biofilm (*Corynebacterium*, 12.6%; *Massilia*, 12.1%; *Actinomyces*, 9.1%; and *Schaalia*, 8.0%). *Prevotella*, *Staphylococcus*, and *Neisseria* were found in similar amounts in these samples as in saliva and the native biofilm.

The 20 most abundant bacterial species identified in these samples are presented in Figure 4 for (a) bacteria found in saliva, (b) bacteria harvested from native biofilms grown on uncoated substrates, and (c) bacteria harvested from biofilms grown on uncoated substrates to which the disinfectant **CHX** had been added. In the analysis by species, more differentiated data is obtained. In particular, some of the species that have a dominant role in saliva do not even appear among the top 20 most abundant bacteria of the native biofilm, while another species from the same genus have a higher abundance in the biofilm than in saliva. For example, the most abundant species in saliva was *Campylobacter concisus* (32.3%), which is also the only bacterium of this genus in the top 20 most abundant species list in saliva. Nonetheless, *C. concisus* does not appear in the untreated biofilm, while *C. ureolyticus* and *C. curvus* have an abundance of 2.0% and 1.3%, respectively. The second most abundant species in saliva, *Gemella sanguinis,* has an abundance of 10.7% in that sample, although does not appear in the native biofilm. Third in line is *Veillonella dispar*, with 7.4% in saliva, and 1.6% in the biofilm. Other *Veillonella* spp. can be found further down the list in saliva: *V. parvula*, *V. rodentium*, and *V. atypica*, but not in the native biofilm. *Streptococcus* sp. LPB0220 is the fourth most abundant species found in saliva, with other *Streptococci* following in positions 7, 12, 13, 18, 19, and 20. Out of these, *Streptococcus salivarius* has the second rank in the top 20 list in the native biofilm, with a relative abundance of 22.9%, while other *Streptococci* (*S. thermophilus*, 2.9%; *S.* sp. LPB0220, 2.4%) follow at rank 9 and 10. *Haemophilus parainfluenzae* (rank 5, 5.5%), *Atopobium parvulum* (rank 9, 3.9%), and *Schaalia odontolytica* (rank 10, 2.4%) and complete the list of the top 10 species in saliva, yet none of them appear in the top 20 list in saliva.

Overall, the native biofilm has a completely different composition of bacterial species, with *Fusobacterium nucleatum* (rank 1, 27.6%) and the aforementioned *Streptococcus salivarius* accounting for half of the present bacteria. *Megasphaera stantonii* (rank 3, 8.9%) and *Megasphaera elsdenii* (rank 4, 8.2) follow in relative abundance. It is interesting to note that there is no such dominance of a few species in the **CHX**-treated biofilm, where *Schaalia odontolytica* (rank 1, 11.9%), *Massilia armeniaca* (rank 2, 9.2%) and *Massilia flava* (rank 3, 7.1%) are followed by species from different genera at low single-digit percentages. Out of the top three, only *Schaalia odontolytica* is found in the top 20 saliva list (rank 10, 2.4%), and none in the native biofilm.

The 20 most abundant genera and species found in biofilms grown on the polymer-coated substrates PSB, PZI, and SMAMP are found in Tables S4 and S5 in the Supporting Information. The most useful way to compare these data with the 20 most abundant genera and species found in the native biofilms was to plot their abundance in CPM side by side (Figure 5 and Figure 6).

Thus, the top 20 most abundant bacterial genera of bacteria harvested from the native biofilms and biofilms grown on polymer-coated the substrates **PSB**, **PZI**, and **SMAMP** are compared in Figure 5. As described above, *Streptococcus* spp., *Fusobacterium* spp., and *Megasphera* spp. are the most abundant bacteria in the native biofilm, followed by *Neisseria* spp., *Tannerella* spp., *Prevoltella* spp., *Campylobacter* spp., and *Veillonella* spp. On **PSB**-coated substrates, these genera also make up the top nine of the list, only in different proportions. Most notably, the share of *Fusobacterium* spp. is reduced from 21.0% to 12.5%, while that of *Campylobacter* spp. increased from 3.8% to 21.1%. The relative abundance of the other top nine genera is quite similar. When comparing the top 20 species list of the native and the **PSB**-grown biofilm (Figure 6 and Table 2), it is found that they have a total of fourteen species in common. *Fusobacterium nucleatum* (rank 2, 13.5%) and *Streptococcus salivarius* (rank 1, 22.8%) were also the most abundant species on **PSB**, although the abundance of *F. nucleatum* (associated with poor oral health) is strongly reduced compared to the native biofilm. Instead, *Campylobacter ureolyticus* takes a share of 11.5% on **PSB** (up from 2%), *Campylobacter curvus* is found with 8.4% abundance (up from 1.3%), and *Campylobacter gracilis*, which does not appear on the list of the native biofilm, has a share of 3.2% on **PSB**.

When comparing the microbial composition of the biofilm on the **SMAMP**-coated substrates and on the **PSB**-coated ones, similar genera are found at the top of the relative abundance list: *Streptococcus*, *Fusobacterium*, *Campylobacter,* and *Megasphaera* are the four most abundant genera, followed by *Veillonella*, *Neisseria*, *Prevotella,* and *Tannerella*. The most striking quantitative differences of **PSB**- and **SMAMP**-coated substrates are the significantly higher abundance of *Fusobacterium* spp. on **SMAMP** (18.8%, compared to 12.5% on **PSB**, and 21% in the native biofilm). In this respect, the biofilm grown on **SMAMP** is in between the bacteria distribution of **PSB** and the uncoated substrate: it has a high amount of *Fusobacterium* spp., like the uncoated substrate, but also a substantial amount of *Campylobacter* spp. Another notable feature of the **SMAMP**-grown biofilm is a significantly higher amount of *Tannerella* spp. (8%, up from 2.5% on **PSB**). Compared to the native biofilm, **SMAMP**-grown biofilms also have a lower amount of *Streptococcus* spp., and possibly *Staphylococcus* spp. (large error bars), *Bacillus* spp., and *Actinomyces* spp., but a higher amount of *Tannerella* spp., *Campylobacter* spp., and *Veillonella* spp.

At the species level (Figure 6, Table 2), it was found that the top 20 list of **SMAMP**-grown and **PSB**-grown biofilms have seventeen species in common, and most of them in a similar amount. On **SMAMP**, *S. salivarius* and *F. nucleatum* are still the most abundant species, although at a more equal proportion to each other than on **PSB**. Moreover, the relative amounts of *M. stantonii*, *M. elsdenii, C. ureolyticus,* and *C. curvus* are at a similar level for both types of coatings. However, *Tannerella* sp. oral taxon HOT-286 ranks in third position in the **SMAMP**-grown biofilm, with 10.8% relative abundance. In the direct comparison of the native biofilm and the biofilm grown on **SMAMP**, a significant reduction of *Acinomyces radicidentis*, *F. nucleatum*, *S. thermophilus*, and *Streptococcus* sp. LPB02 20 is observed on **SMAMP**, while the amounts of *C. ureolyticus*, *C. curvus*, *Fusobacterium pseudoperiodonticum*, and *Tanerella* sp. oral taxon HOT-286 are significantly increased.

The biofilm grown on **PZI**-coated surfaces appears more similar to the native biofilm on the genera level than the other coated substrates (Figure 5). Both the order of abundance of the top four genera are the same in both samples, and their relative amounts are similar. The other top twelve genera follow in a slightly different order yet with still comparable amounts, except for *Veillonella*, which increases from 3.7% in the native biofilm to 6.4% on the **PZI**-grown biofilm. At the species level, this finding is confirmed, with *F. nucleatum*, *S. salivarius*, *M. stantonii*, and *M. elsdenii* found as the top four species and at comparable relative abundance in **PZI**-grown and native biofilms (Table 2, Figure 6). Other species that have similar levels in both sample types are *F. pseudoperiodonticum*, *N. subflavia*, *Prevotella dentalis*, *C. ureolyticus*, *C. curvus*, and others. Notably, **PZI**-grown biofilms contain 3.8% of *Neisseria mucosa*, 1.7% of *Veillonella parvula*, and 1.4% *Veillonella rodentium*, which are all not among the top 20 species in the native biofilm. However, all three species appear in the biofilms grown on **PSB** and **SMAMP**, and both *Veillonella* spp. were present in significant amounts in saliva.

## 4. Discussion

Three poly(oxanorbornene)-based polymer coatings with different bioactivity profile were chosen for this study to evaluate their effects on the microbial composition of biofilms formed by total human salivary bacteria: The poly(oxanorbornene)-based poly(sulfobetaine) (**PSB**), the polycationic (**SMAMP**) and the third coating is a poly(carboxyzwitterion) (**PZI**). After 72 h, the biofilms were harvested and analyzed by 16S RNA sequencing.

Using total salivary bacteria to evaluate the biofilm formation on different material surface has been shown to be a meaningful model, since new materials cannot be tested in vivo within the oral cavity and since oral human salivary microbiota has a high diversity, which reflects a worst case of biomaterial-associated infections [73]. The bacterial biofilm composition was analyzed by sequencing the 16S ribosomal RNA as previously published [74], using full length sequencing of the 16S rRNA gene with nanopore sequencing, which allows for very sensitive differentiation of bacterial composition in the analyzed samples. Synthesis of cDNA and hence 16S rRNA sequencing was chosen over 16S rDNA sequencing as this approach is better suited for evaluating samples of living bacteria [74]. Using this technique is meaningful to evaluate the effects of polymer coatings on adherent bacteria.

With exception of *Bacillus* spp. and *Staphylococcus* spp., all of these abundant bacteria have been described in the in-situ oral biofilm [1,5,19]. In a state of oral health, there is an equilibrium of these different bacteria in the oral cavity [14]. Deviations of this equilibrium have been associated with various oral diseases. For example, a high abundance of *Fusobacterium* spp., *Tannerella* spp., *Prevotella* spp., and *Campylobacter* spp. have been associated with poor periodontal oral health or peri-implantitis [5,12,13,75]. An increase of the abundance of *Streptococcus* spp. and *Veillonella* spp. is also an indication of poor oral health regarding caries development [1,8]. The detection of *Staphylococcus* spp. and *Bacillus* spp. in the native biofilm may be related to food consumption, since foodborne bacteria such as *Enterococcus* spp. have been shown to be able to integrate in oral biofilms in situ [76]. However, it has been shown that *Staphylococcus aureus* and *Staphylococcus epidermidis* can be associated with peri-implantitis of titanium oral implants [77,78].

In the biofilm grown in the presence of **CHX**, the amount of recovered bacterial RNA was overall more than one order of magnitude lower than in the native biofilm, thus confirming the well-document activity of **CHX** against bacteria [79,80]. Among the bacteria detected in the biofilm grown in the presence of CHX, *Schaalia* spp. have been isolated from tongue biofilms [81], whereas *Massilia* spp. are a non-oral bacteria that have been isolated from environment (air and soil), tobacco and the blood of an immunocompromised patient, as well as from an otitis media infections [82,83]. Since there is evidence that bacteria may develop resistance towards chlorhexidine digluconate [84], it cannot be excluded that members of these taxa are less sensitive towards **CHX** and hence overgrew other typical oral biofilm bacteria.

The results from the total extractable RNA are in strong agreement with previous results of protein adhesion studies on the polymer-coated substrates [63,64]. **PSB** and **PZI**—which are known to be protein-repellent—also reduced the amount of extractable RNA (and thus the number of living bacteria) by 90%, compared to the uncoated substrate. While **PZI** has an additional intrinsic antimicrobial activity, **PSB** does not, whereby this reduction seems to be an effect of reduced bacterial adhesion on the surface, rather than an effect of antimicrobial activity. On the other hand, **SMAMP** is mildly antimicrobial and polycationic, and thus protein-adhesive [63,64,65]. Consequently, its reduction of the extractable RNA is only 40%. This indicates that bacteria can adhere on this surface, but some of the adhering bacteria are killed, so that the overall amount able to initiate biofilm formation is reduced. When comparing the native biofilm to all three coating types, it was found that **SMAMP** caused a higher relative abundance of *Campylobacter* spp., *F. pseudoperiodonticum* and *Tanerella* sp. oral taxon HOT-286), while other bacteria were diminished (e.g., two each of the *Streptococcus* spp. and *Prevotella* spp.). Importantly, the **SMAMP** coating also significantly reduced the relative abundance of the top two biofilm species, the less pathogenic *Fusobacterium nucleatum* and the caries-associated *Streptococcus salivarius*.

For the protein-repellent **PSB** coating, the error bars of the data were substantially larger than for the other sample types in most cases. This can be explained by the pH-independent anti-adhesive properties of this material, which slowed down the initial bacterial adhesion dramatically, as seen in the RNA amount. However, once the initial adhesion (of bacteria, debris of dead bacteria, or other biomolecules) has occurred, the bacteria can also grow exponentially on this layer. When this process differs on each sample, e.g., due to slightly different environmental conditions, this could result in larger errors compared to the potentially adhesive **SMAMP**, **PZI**, and uncoated substrates. Interestingly, there are some bacteria that show bacterial counts in the order **PSB** > **SMAMP** >/≈ **PZI** (e.g., *V. dispar*) while others show the exact opposite trend (*A. radicidentis*, *F. nucleatum*, *N. subflava*), and yet others show no trend at all. This indicates a complex interplay between the surface features and the microbiological features of the individual bacterial species, particularly in a complex mixture like saliva, which is not yet fully understood at present.

*Campylobacter* spp. seem able to colonize the protein-repellent **PSB** coating more easily than, for example, *Fusobacterium nucleatum*, whose abundance is reduced by almost half on **PSB** coating. On the other hand, the relative abundance *Streptococcus salivarius* (associated with caries) [1,8] and the two *Megasphaera* species *M. stantonii* and *M. elsdenii*, seems less affected (although the **PSB** error bars are large for these species), thus indicating no decisive preference for either substrate. The proportions of certain *Veillonella* spp. also indicate that the polymer coatings facilitate the adhesion of these three species, irrespective of their type of bioactivity, albeit at low levels. This stresses that some microbial members of the biofilms are not affected by any of the coatings. The microbiome analysis by means of 16S rRNA sequencing used here has been accepted and recommended to deliver a realistic composition of microbial niches, thereby including those species that cannot be cultivated using culture techniques. However, the 16S rRNA number is also dependent on the number of active bacteria, which includes more ribosomes and could thus lead to false positive abundance values of certain bacterial cells. Nevertheless, one advantage of this technique is that the revealed abundance number reflects the live as opposed to the dead bacteria. This is an important aspect when the antimicrobial activity of polymers on biofilm formation has to be investigated.

In summary, the tested materials modified the microbial composition of the biofilm formed by total salivary bacteria in different ways. From a clinical and ecological perspective, alternative treatment methods of oral biofilms in which the oral microbiota are modulated towards the healthy equilibrium are required. In this context, the present results are encouraging, since some bacterial members such as *Tannerella forsythia*, *Prevotella denticola,* and *Fusobacterium nucleatum* associated with poor oral health were reduced by the coatings, whereas other species belonging to the physiological resident flora such as some *Streptococcus* species and *Neisseria mucosa* were not affected. Hence, the present results encourage evaluating the clinical relevance of the tested coatings *in situ*.

## 5. Conclusions

The surface coatings **PSB**, **PZI,** and **SMAMP** analyzed in this study exhibited differential ability to reduce biofilm formation upon challenge with a complex bacterial mixture obtained from saliva. Although none of them prevented the formation of the biofilm, there was a drastic reduction in the overall amount of surface-attached bacteria, as well as the microbial composition of biofilm. Notably, the two protein-repellent coatings **PSB** and **PZI** reduced the amount of biofilm by one order of magnitude after 72 h, while the mildly antimicrobial coating **SMAMP** only had a 40% reduction. This is an indication that coatings with protein repellency may be more efficient in reducing biofilm formation in situations where there is a high load of bacteria—such as in the oral cavity—than antimicrobial coatings. Additionally, for maintaining oral health, eliminating all bacteria in the oral cavity is not desired due to the existing synergism between the host and the microbiome. Thus, the ability to induce microbiome modification could be a much more desirable property than the ability to eliminate biofilms. Especially the reduction of some bacterial species associated with poor oral health such as *Tannerella forsythia* (observed for **PSB** and **SMAMP**), *Fusobacterium nucleatum* (also observed for **PSB** and **SMAMP**), and *Prevotella denticola* (observed for all coatings) may positively modulate the oral biofilm also in situ, which should be tested in further studies. Additionally, due to the association of *Staphylococcus* spp. with peri-implantitis on titanium-based oral implants and possible antibiotic resistance of these species, the lower abundance of these bacteria with the coatings is encouraging to further evaluate the biofilm formation ability of *Staphylococcus aureus* and *Staphylococcus epidermidis* on all tested coatings.

Further studies into such modulation of biofilm composition by surface coatings could enable developing surfaces that provide both a strong reduction in biofilm formation and a modulation of biofilm composition that favors non-harmful bacteria over pathogenic species.

## Figures and Tables

**Table 2:** Relative abundance of bacteria (in percent), found in the native biofilm, and the biofilms formed on **PSB**-, **PZI**-, and **SMAMP**-coated substrates, listed by species. Standard deviations of the data are found in Tables S2–S5 in the Supporting Information.

Species	Native	PSB	PZI	SMAMP
*Fusobacterium nucleatum*	27.6	13.5	27.3	18.0
*Streptococcus salivarius*	22.9	21.8	26.1	18.8
*Megasphaera stantonii*	8.9	8.4	7.2	7.3
*Megasphaera elsdenii*	8.2	7.9	7.2	7.0
*Prevotella dentalis*	4.0	4.5	2.7	1.5
*Tannerella* sp. oral taxon HOT-286	4.0	3.7	4.8	10.8
*Tannerella forsythia*	3.3	n.d.	1.2	n.d.
*Neisseria subflava*	3.3	3.0	2.1	1.2
*Streptococcus thermophilus*	2.9	2.0	2.2	1.5
*Streptococcus* sp. LPB0220	2.4	1.3	0.9	n.d.
*Campylobacter ureolyticus*	2.0	11.5	3.1	8.9
*Veillonella dispar*	1.6	2.5	2.0	n.d.
*Staphylococcus schleiferi*	1.6	0.8	1.2	1.0
*Campylobacter curvus*	1.3	8.4	2.1	5.9
*Fusobacterium pseudoperiodonticum*	1.3	1.9	1.3	3.7
*Luteimonas* sp. Gr-4	1.0	n.d.	n.d.	n.d.
*Slackia heliotrinireducens*	1.0	n.d.	n.d.	n.d.
*Erysipelothrix larvae*	0.9	n.d.	n.d.	n.d.
*Prevotella denticola*	0.9	n.d.	n.d.	n.d.
*Actinomyces radicidentis*	0.9	n.d.	n.d.	n.d.
*Neisseria mucosa*	n.d.	1.5	3.8	3.7
*Veillonella parvula*	n.d.	1.4	1.7	1.1
*Veillonella rodentium*	n.d.	1.2	1.4	0.9
*Porphyromonas gingivalis*	n.d.	n.d.	1.0	n.d.
*Streptococcus gordonii*	n.d.	n.d.	0.7	n.d.
*Campylobacter gracilis*	n.d.	3.2	n.d.	3.0
*Streptococcus parasanguinis*	n.d.	n.d.	n.d.	2.3
*Erysipelothrix larvae*	n.d.	n.d.	n.d.	0.8
*Lachnoanaerobaculum umeaense*	n.d.	n.d.	n.d.	0.8
*Prevotella denticola*	n.d.	0.8	n.d.	n.d.
*Campylobacter concisus*	n.d.	0.8	n.d.	n.d.

## Data Availability

The data are available on request from the authors.

## Supplementary Materials

The following are available online at https://www.mdpi.com/article/10.3390/microorganisms9071427/s1, Table S1: Total RNA extracted from biofilm samples grown on PSB, PZI, and SMAMP, uncoated substrates (NegCtrol), and uncoated substrates grown in the presence of chlorhexidine digluconate (CHX). Data is as concentration (in ng/µl), total mass of extracted RNA (in ng), and relative percentage compared to the mean of the uncoated samples (NegCtrol), i.e. the natively biofilm grown., Table S2: Abundance of bacteria (in counts per million, CPM, and percent), found in (a) saliva, (b) the native biofilm formed on uncoated substrates, and (c) the biofilms formed on uncoated substrates in the presence of CHX, listed as Top 20 genera. STD = Standard Deviation. It should be kept in mind that the amount of 16S rRNA also correlates to a high number of ribosomes, which in turn correlates with the activity of bacteria, since active bacterial cells contain a higher number of ribosomes than inactive ones, Table S3: Abundance of bacteria (in counts per million, CPM, and percent), found in (a) saliva, (b) the native biofilm formed on uncoated substrates, and (c) the biofilms formed on uncoated substrates in the presence of CHX, listed as Top 20 species. STD = Standard Deviation. It should be kept in mind that the amount of 16S rRNA also correlates to a high number of ribosomes, which in turn correlates with the activity of bacteria, since active bacterial cells contain a higher number of ribosomes than inactive ones, Table S4: Abundance of bacteria (in counts per million, CPM, and percent), found in (a) biofilms grown on PSB, (b) biofilms grown on PZI, and (c) biofilms grown on SMAMP, listed as Top 20 genera. STD = Standard Deviation. It should be kept in mind that the amount of 16S rRNA also correlates to a high number of ribosomes, which in turn correlates with the activity of bacteria, since active bacterial cells contain a higher number of ribosomes than inactive ones, Table S5: Abundance of bacteria (in counts per million, CPM, and percent), found in (a) biofilms grown on PSB, (b) biofilms grown on PZI, and (c) biofilms grown on SMAMP, listed as Top 20 species. STD = Standard Deviation. It should be kept in mind that the amount of 16S rRNA also correlates to a high number of ribosomes, which in turn correlates with the activity of bacteria, since active bacterial cells contain a higher number of ribosomes than inactive ones, Table S6: About 950,000 to 1,500,000 sequencing reads passing the ONT Q7 quality score were obtained for each sample, and of these >95% in each sample mapped to bacterial sequences as per the centrifuge results, Figure S1: Z-section galleries of representative confocal laser scanning microscopic (CLSM) images depicting the ex vivo biofilm formation (3 days) after live/dead staining. The panels illustrate the live (green) and dead (red) microbial populations of biofilms grown on the PSB coating. The multiple Z-sections in the panels were generated by vertical sectioning at 2.0-µm intervals through the sample above the PSB surface. Bars, 20 µm.”, Figure S2: Alpha (A) and beta (B) diversity measures were calculated using the R package vegan (Community Ecology Package, R package version 2.5-7., https://CRAN.R-project.org/package=vegan, published by J. Oksanen, F. Guillaume Blanchet, Michael Friendly, Roeland Kindt, Pierre Legendre, Dan McGlinn, Peter R. Minchin, R. B. O’Hara, Gavin L. Simpson, Peter Solymos, M. Henry H. Stevens, Eduard Szoecs and Helene Wagner 2020 accessed on 1 March 2021) and visualized using the ggplot and pheatmap packages (Raivo Kolde 2019: R package version 1.0.12. https://CRAN.R-project.org/package=pheatmap accesed on 1 March 2021).
