# Supplementary material for: How Do Polymer Coatings Affect the Growth and Bacterial Population of a Biofilm Formed by Total Human Salivary Bacteria?—A Study by 16S-RNA Sequencing
Source: Microorganisms. 2021 Jul 1;9(7):1427. doi: 10.3390/microorganisms9071427 (PMC8304871; doi:10.3390/microorganisms9071427)

microorganisms

MDPI

Supporting Information

Type of the Paper (Article)

How Do Polymer Coatings Affect the Growth and Bacterial Population of a Biofilm Formed by Total Human Salivary Bacteria? – A Study by 16S-RNA Sequencing

Ali Al-Ahmad[1], Kira Wollensak[1,2], Sybille Rau[1], Diana Lorena Guevara Solarte[1], Stefan Paschke[2], Karen Lienkamp[2,3], Ori Staszewski[4]

[1] Department of Operative Dentistry and Periodontology, Medical Center, University of Freiburg, Faculty of Medicine, University of Freiburg, Hugstetter Strasse 55, 79106 Freiburg, Germany; ali.al-ahmad@uniklinik-freiburg.de, kirawollensak@yahoo.de, sibylle.rau@uniklinik-freiburg.de, diana.lorena.guevara.solarte@uniklinik-freiburg.de

[2] Bioactive Polymer Synthesis and Surface Engineering Group, Department of Microsystems Engineering (IMTEK) and Freiburg Center for Interactive Materials and Bioinspired Technologies (FIT), University of Freiburg, Georges-Kohler-Allee 105, 79110 Freiburg, Germany; stefan.paschke@imtek.uni-freiburg.de

[3] Institut für Materialwissenschaft und Werkstoffkunde, Universität des Saarlandes, Campus, 66123 Saarbrücken, Germany; karen.lienkamp@uni-saarland.de

[4] Institute of Neuropathology, Medical Center of the University of Freiburg, Faculty of Medicine, University of Freiburg, 79106 Freiburg, Germany; ori.staszewski@uniklinik-freiburg.de

* Correspondence: ali.al-ahmad@uniklinik-freiburg.de; Tel.: +49 761 27048940

Table of Contents:

Table S1: Total RNA extracted from biofilm samples grown on **PSB**, **PZI**, and **SMAMP**, uncoated substrates (**NegCtrol**), and uncoated substrates grown in the presence of chlorhexidine digluconate (**CHX**). Data is as concentration (in ng/µl), total mass of extracted RNA (in ng), and relative percentage compared to the mean of the uncoated samples (**NegCtrol**), i.e. the natively biofilm grown.

Table S2: Abundance of bacteria (in counts per million, CPM, and percent), found in a) saliva, b) the native biofilm formed on uncoated substrates, and c) the biofilms formed on uncoated substrates in the presence of **CHX**, listed as Top 20 genera. STD = Standard Deviation. It should be kept in mind that the amount of 16S rRNA also correlates to a high number of ribosomes, which in turn correlates with the activity of bacteria, since active bacterial cells contain a higher number of ribosomes than inactive ones.

Table S3: Abundance of bacteria (in counts per million, CPM, and percent), found in a) saliva, b) the native biofilm formed on uncoated substrates, and c) the biofilms formed on uncoated substrates in the presence of **CHX**, listed as Top 20 species. STD = Standard Deviation. It should be kept in mind that the amount of 16S rRNA also correlates to a high number of ribosomes, which in turn correlates with the activity of bacteria, since active bacterial cells contain a higher number of ribosomes than inactive ones.

microorganisms

Table S4: Abundance of bacteria (in counts per million, CPM, and percent), found in a) biofilms grown on **PSB**, b) biofilms grown on **PZI**, and c) biofilms grown on **SMAMP**, listed as Top 20 genera. STD = Standard Deviation. It should be kept in mind that the amount of 16S rRNA also correlates to a high number of ribosomes, which in turn correlates with the activity of bacteria, since active bacterial cells contain a higher number of ribosomes than inactive ones.

Table S5: Abundance of bacteria (in counts per million, CPM, and percent), found in a) biofilms grown on **PSB**, b) biofilms grown on **PZI**, and c) biofilms grown on **SMAMP**, listed as Top 20 species. STD = Standard Deviation. It should be kept in mind that the amount of 16S rRNA also correlates to a high number of ribosomes, which in turn correlates with the activity of bacteria, since active bacterial cells contain a higher number of ribosomes than inactive ones.

Table S1: Total RNA extracted from biofilm samples grown on **PSB**, **PZI**, and **SMAMP**, uncoated substrates (**NegCtrol**), and uncoated substrates grown in the presence of chlorhexidine digluconate (**CHX**). Data is as concentration (in ng/µl), total mass of extracted RNA (in ng), and relative percentage compared to the mean of the uncoated samples (**NegCtrol**), i.e. the natively biofilm grown.

Sample	ng/µl	ng	% of NegCtrl
**NegCtrl 1**	15.1	211.8	106.6
**NegCtrl 2**	13.3	186.1	93.6
**NegCtrl 3**	12.1	169.4	85.3
**NegCtrl 4**	16.3	227.5	114.5
**SMAMP 1**	7.4	103.3	52.0
**SMAMP 2**	6.5	91.3	45.9
**SMAMP 3**	7.5	104.4	52.6
**SMAMP 4**	13.4	187.6	94.4
**SMAMP 5**	7.4	103.9	52.3
**PZI 1**	1.6	21.8	11.0
**PZI 2**	1.0	13.7	6.9
**PZI 3**	1.1	15.8	8.0
**PZI 4**	0	0	0
**PZI 5**	1.1	15.3	7.7
**PSB 1**	1.5	21.6	10.9
**PSB 2**	2.0	28.3	14.2
**PSB 3**	0.9	12.4	6.1
**PSB 4**	1.5	20.6	10.4
**CHX**	0.6	8.5	4.3

Table S2: Abundance of bacteria (in counts per million, CPM, and percent), found in a) saliva, b) the native biofilm formed on uncoated substrates, and c) the biofilms formed on uncoated substrates in the presence of **CHX**, listed as Top 20 genera. STD = Standard Deviation. It should be kept in mind that the amount of 16S rRNA also correlates to a high number of ribosomes, which in turn correlates with the activity of bacteria, since active bacterial cells contain a higher number of ribosomes than inactive ones.

**a) Saliva**				
**Genus**	**CPM Mean**	**CPM STD**	**% Mean**	**% STD**
Streptococcus	298653.96	13058.34	29.87	1.31
Campylobacter	202530.06	61853.40	20.25	6.19
Veillonella	164369.75	62496.87	16.44	6.25
Gemella	67682.07	46469.58	6.77	4.65

Prevotella	34458.58	30544.07	3.45	3.05
Haemophilus	32488.68	10128.32	3.25	1.01
Fusobacterium	31683.54	9174.90	3.17	0.92
Neisseria	28694.34	19290.07	2.87	1.93
Schaalia	20082.88	2454.18	2.01	0.25
Actinomyces	14874.74	6317.08	1.49	0.63
Atopobium	14507.75	14025.36	1.45	1.40
Bacillus	9462.43	2549.41	0.95	0.25
Selenomonas	6566.19	3007.45	0.66	0.30
Rothia	6466.50	1436.57	0.65	0.14
Staphylococcus	5781.25	716.37	0.58	0.07
Lactobacillus	5213.19	1120.81	0.52	0.11
Corynebacterium	3970.43	3429.50	0.40	0.34
Enterococcus	3732.74	1439.90	0.37	0.14
Erysipelothrix	2771.61	2110.45	0.28	0.21
Megasphaera	2090.94	1038.76	0.21	0.10

b) Native

Genus	CPM Mean	CPM STD	% Mean	% STD
Streptococcus	316633.26	28601.86	31.66	2.86
Fusobacterium	187175.01	37219.62	18.72	3.72
Megasphaera	106519.42	23815.29	10.65	2.38
Neisseria	46108.91	44862.71	4.61	4.49
Tannerella	42883.65	34077.78	4.29	3.41
Prevotella	39814.53	36564.89	3.98	3.66
Campylobacter	33569.54	22700.58	3.36	2.27
Veillonella	32888.34	11113.57	3.29	1.11
Staphylococcus	11871.88	5037.34	1.19	0.50
Bacillus	10803.27	1177.49	1.08	0.12
Selenomonas	9247.11	2669.07	0.92	0.27
Actinomyces	9000.47	3912.34	0.90	0.39
Erysipelothrix	7806.01	2268.61	0.78	0.23
Luteimonas	6163.59	7338.72	0.62	0.73
Slackia	5933.55	2958.09	0.59	0.30
Dialister	5791.48	1041.49	0.58	0.10
Atopobium	4728.39	1154.16	0.47	0.12
Olsenella	4550.73	2315.17	0.46	0.23
Schaalia	4362.96	1258.85	0.44	0.13
Gemella	4287.27	1423.55	0.43	0.14

c) CHX

Genus	CPM Mean	CPM STD	% Mean	% STD
Streptococcus	21.01	n.a.	21.01	n.a.
Corynebacterium	10.47	n.a.	10.47	n.a.
Massilia	10.06	n.a.	10.06	n.a.
Actinomyces	7.55	n.a.	7.55	n.a.
Schaalia	6.62	n.a.	6.62	n.a.
Prevotella	4.01	n.a.	4.01	n.a.
Staphylococcus	2.79	n.a.	2.79	n.a.
Neisseria	2.56	n.a.	2.56	n.a.
Rothia	2.14	n.a.	2.14	n.a.
Lautropia	2.12	n.a.	2.12	n.a.
Porphyromonas	2.03	n.a.	2.03	n.a.
Erysipelothrix	1.87	n.a.	1.87	n.a.
Selenomonas	1.87	n.a.	1.87	n.a.

Streptomyces	1.78	n.a.	1.78	n.a.
Brevundimonas	1.68	n.a.	1.68	n.a.
Bacillus	1.43	n.a.	1.43	n.a.
Bradyrhizobium	0.90	n.a.	0.90	n.a.
Fusobacterium	0.79	n.a.	0.79	n.a.
Gemella	0.78	n.a.	0.78	n.a.
Atopobium	0.78	n.a.	0.78	n.a.

Table S3: Abundance of bacteria (in counts per million, CPM, and percent), found in a) saliva, b) the native biofilm formed on uncoated substrates, and c) the biofilms formed on uncoated substrates in the presence of **CHX**, listed as Top 20 species. STD = Standard Deviation. It should be kept in mind that the amount of 16S rRNA also correlates to a high number of ribosomes, which in turn correlates with the activity of bacteria, since active bacterial cells contain a higher number of ribosomes than inactive ones.

**a) Saliva**				
**Species**	**CPM Mean**	**CPM STD**	**% Mean**	**% STD**
Campylobacter concisus	243034.20	57565.27	24.30	5.76
Gemella sanguinis	80512.50	53728.22	8.05	5.37
Veillonella dispar	55877.49	30950.55	5.59	3.10
Streptococcus sp. LPB0220	48460.60	18826.01	4.85	1.88
Haemophilus parainfluenzae	41176.31	15305.59	4.12	1.53
Veillonella parvula	36101.70	9000.34	3.61	0.90
Streptococcus sp. A12	33867.45	25390.49	3.39	2.54
Veillonella rodentium	29574.61	10443.03	2.96	1.04
Atopobium parvulum	18811.03	17446.63	1.88	1.74
Schaalia odontolytica	17907.65	2575.89	1.79	0.26
Prevotella sp. oral taxon 299	17656.55	24036.68	1.77	2.40
Streptococcus koreensis	17615.67	9148.69	1.76	0.91
Streptococcus sp. I-P16	17566.03	13629.89	1.76	1.36
Prevotella melaninogenica	17467.38	13818.00	1.75	1.38
Fusobacterium pseudoperiodonticum	15987.26	3072.26	1.60	0.31
Veillonella atypica	13566.61	4695.02	1.36	0.47
Neisseria subflava	13364.14	9965.92	1.34	1.00
Streptococcus salivarius	12703.52	8865.47	1.27	0.89
Streptococcus pneumoniae	11627.69	5475.84	1.16	0.55
Streptococcus sp. oral taxon 431	10426.36	4820.18	1.04	0.48

**b) Native**				
**Species**	**CPM Mean**	**CPM STD**	**% Mean**	**% STD**
Fusobacterium nucleatum	201697.88	44677.38	20.17	4.47
Streptococcus salivarius	167185.28	44236.34	16.72	4.42
Megasphaera stantonii	64882.66	19192.01	6.49	1.92
Megasphaera elsdenii	60280.24	10042.85	6.03	1.00
Prevotella dentalis	29272.85	28919.48	2.93	2.89
Tannerella sp. oral taxon HOT-286	29227.20	24514.84	2.92	2.45
Tannerella forsythia	24046.18	47418.83	2.40	4.74
Neisseria subflava	23884.48	24315.76	2.39	2.43
Streptococcus thermophilus	21101.02	1692.00	2.11	0.17
Streptococcus sp. LPB0220	17507.70	5630.60	1.75	0.56
Campylobacter ureolyticus	14987.13	10940.48	1.50	1.09
Veillonella dispar	12046.56	4916.29	1.20	0.49
Staphylococcus schleiferi	11557.77	5093.23	1.16	0.51
Campylobacter curvus	9809.15	7004.67	0.98	0.70

Fusobacterium pseudoperiodonticum	9788.53	3869.59	0.98	0.39
Luteimonas sp. Gr-4	7552.74	8932.71	0.76	0.89
Slackia heliotrinireducens	7272.81	3535.70	0.73	0.35
Erysipelothrix larvae	6907.04	2090.77	0.69	0.21
Prevotella denticola	6349.18	6035.12	0.63	0.60
Actinomyces radicidentis	6251.42	3857.25	0.63	0.39

**c) CHX**				
**Species**	**CPM Mean**	**CPM STD**	**% Mean**	**% STD**
Schaalia odontolytica	56431.94	n.a.	5.64	n.a.
Massilia armeniaca	43702.81	n.a.	4.37	n.a.
Massilia flava	33687.56	n.a.	3.37	n.a.
Lautropia mirabilis	28406.33	n.a.	2.84	n.a.
Prevotella melaninogenica	24918.81	n.a.	2.49	n.a.
Streptococcus sanguinis	24449.29	n.a.	2.44	n.a.
Rothia mucilaginosa	23693.40	n.a.	2.37	n.a.
Actinomyces naeslundii	23493.61	n.a.	2.35	n.a.
Actinomyces radicidentis	22014.02	n.a.	2.20	n.a.
Schaalia meyeri	21756.50	n.a.	2.18	n.a.
Streptomyces sp. SCSIO 03032	21525.63	n.a.	2.15	n.a.
Porphyromonas gingivalis	21097.18	n.a.	2.11	n.a.
Streptococcus salivarius	18466.56	n.a.	1.85	n.a.
Erysipelothrix larvae	18052.54	n.a.	1.81	n.a.
Neisseria mucosa	17014.72	n.a.	1.70	n.a.
Staphylococcus schleiferi	16263.27	n.a.	1.63	n.a.
Selenomonas sp. oral taxon 136	16152.27	n.a.	1.62	n.a.
Streptococcus parasanguinis	14562.79	n.a.	1.46	n.a.
Streptococcus thermophilus	14006.70	n.a.	1.40	n.a.
Streptococcus sp. LPB0220	13078.77	n.a.	1.31	n.a.

Table S4: Abundance of bacteria (in counts per million, CPM, and percent), found in a) biofilms grown on **PSB**, b) biofilms grown on **PZI**, and c) biofilms grown on **SMAMP**, listed as Top 20 genera. STD = Standard Deviation. It should be kept in mind that the amount of 16S rRNA also correlates to a high number of ribosomes, which in turn correlates with the activity of bacteria, since active bacterial cells contain a higher number of ribosomes than inactive ones.

**a) PSB**				
**Genus**	**CPM Mean**	**CPM STD**	**% Mean**	**% STD**
Streptococcus	265594.50	81644.39	26.56	8.16
Campylobacter	198685.49	79362.69	19.87	7.94
Fusobacterium	118246.36	52794.69	11.82	5.28
Megasphaera	108818.28	53636.62	10.88	5.36
Veillonella	58971.81	21884.42	5.90	2.19
Neisseria	57819.28	75321.98	5.78	7.53
Prevotella	48485.56	14916.20	4.85	1.49
Tannerella	23453.89	10048.99	2.35	1.00
Treponema	8395.22	4023.67	0.84	0.40
Bacillus	7734.32	2490.42	0.77	0.25
Selenomonas	7115.98	4096.00	0.71	0.41
Staphylococcus	7012.64	3555.46	0.70	0.36
Erysipelothrix	5930.60	1931.54	0.59	0.19
Dialister	5367.26	3061.18	0.54	0.31
Slackia	4177.49	4734.10	0.42	0.47
Capnocytophaga	3804.51	4087.29	0.38	0.41

Actinomyces	3683.44	1424.23	0.37	0.14
Porphyromonas	3565.28	1219.20	0.36	0.12
Olsenella	3384.43	3992.79	0.34	0.40
Gemella	2411.59	1257.08	0.24	0.13

b) PZI

Genus	CPM Mean	CPM STD	% Mean	% STD
Streptococcus	312564.78	25573.85	31.26	2.56
Fusobacterium	207524.82	45508.91	20.75	4.55
Megasphaera	99710.25	20991.16	9.97	2.10
Neisseria	67259.26	55640.46	6.73	5.56
Veillonella	59921.59	17969.10	5.99	1.80
Campylobacter	55033.42	31434.41	5.50	3.14
Tannerella	39875.61	18402.07	3.99	1.84
Prevotella	29447.34	11419.90	2.94	1.14
Staphylococcus	9089.19	3394.30	0.91	0.34
Bacillus	8179.89	1356.71	0.82	0.14
Selenomonas	7716.39	2385.99	0.77	0.24
Actinomyces	6706.52	4479.26	0.67	0.45
Porphyromonas	6666.39	8219.77	0.67	0.82
Erysipelothrix	6380.08	2176.81	0.64	0.22
Dialister	5474.05	1586.33	0.55	0.16
Slackia	3122.74	1258.16	0.31	0.13
Rothia	2966.57	2035.97	0.30	0.20
Eikenella	2823.39	540.41	0.28	0.05
Tetragenococcus	2639.59	762.81	0.26	0.08
Bacteroides	2561.54	2662.53	0.26	0.27

c) SMAMP

Genus	CPM Mean	CPM STD	% Mean	% STD
Streptococcus	237743.35	26999.99	23.77	2.70
Fusobacterium	177287.15	32570.35	17.73	3.26
Campylobacter	162427.64	66013.08	16.24	6.60
Megasphaera	102910.17	16333.80	10.29	1.63
Tannerella	75675.60	15826.68	7.57	1.58
Neisseria	56166.63	30690.84	5.62	3.07
Veillonella	46971.14	12631.36	4.70	1.26
Prevotella	19286.51	6632.46	1.93	0.66
Selenomonas	8480.92	1734.02	0.85	0.17
Staphylococcus	8239.61	2098.15	0.82	0.21
Erysipelothrix	7910.86	3407.19	0.79	0.34
Bacillus	7340.60	685.10	0.73	0.07
Lachnoanaerobaculum	5428.10	1737.52	0.54	0.17
Dialister	5308.95	641.20	0.53	0.06
Atopobium	4667.43	2045.56	0.47	0.20
Capnocytophaga	4261.01	5426.32	0.43	0.54
Actinomyces	4028.15	1522.07	0.40	0.15
Slackia	3803.65	1846.80	0.38	0.18
Olsenella	2905.63	1265.99	0.29	0.13
Tetragenococcus	2838.57	1126.21	0.28	0.11

microorganisms MDPI

Table S5: Abundance of bacteria (in counts per million, CPM, and percent), found in a) biofilms grown on **PSB**, b) biofilms grown on **PZI**, and c) biofilms grown on **SMAMP**, listed as Top 20 species. STD = Standard Deviation. It should be kept in mind that the amount of 16S rRNA also correlates to a high number of ribosomes, which in turn correlates with the activity of bacteria, since active bacterial cells contain a higher number of ribosomes than inactive ones.

a) PSB

Species	CPM Mean	CPM STD	% Mean	% STD
Streptococcus salivarius	173280.61	84957.71	17.33	8.50
Fusobacterium nucleatum	107372.78	61657.44	10.74	6.17
Campylobacter ureolyticus	91127.00	40955.75	9.11	4.10
Megasphaera stantonii	67148.17	31990.75	6.71	3.20
Campylobacter curvus	66476.42	30465.27	6.65	3.05
Megasphaera elsdenii	63067.95	33766.82	6.31	3.38
Prevotella dentalis	35491.59	13921.09	3.55	1.39
Tannerella sp. oral taxon HOT-286	29360.93	13421.85	2.94	1.34
Campylobacter gracilis	25427.38	15465.63	2.54	1.55
Neisseria subflava	24153.68	43798.98	2.42	4.38
Veillonella dispar	19560.27	10960.46	1.96	1.10
Streptococcus thermophilus	16094.37	4529.98	1.61	0.45
Fusobacterium pseudoperiodonticum	15037.92	6136.79	1.50	0.61
Neisseria mucosa	12018.73	15115.77	1.20	1.51
Veillonella parvula	10923.53	2524.97	1.09	0.25
Streptococcus sp. LPB0220	10375.91	9551.32	1.04	0.96
Veillonella rodentium	9242.57	2483.41	0.92	0.25
Staphylococcus schleiferi	6659.69	3259.97	0.67	0.33
Prevotella denticola	6543.12	2144.30	0.65	0.21
Campylobacter concisus	6286.42	1970.64	0.63	0.20

b) PZI

Species	CPM Mean	CPM STD	% Mean	% STD
Fusobacterium nucleatum	215937.68	51565.22	21.59	5.16
Streptococcus salivarius	205957.66	13785.24	20.60	1.38
Megasphaera stantonii	56966.06	14114.85	5.70	1.41
Megasphaera elsdenii	56478.69	12560.91	5.65	1.26
Tannerella sp. oral taxon HOT-286	38194.94	8229.52	3.82	0.82
Neisseria mucosa	29639.62	52183.58	2.96	5.22
Campylobacter ureolyticus	24100.65	14212.72	2.41	1.42
Prevotella dentalis	21313.02	7829.72	2.13	0.78
Streptococcus thermophilus	17681.01	3802.28	1.77	0.38
Neisseria subflava	16617.37	11621.04	1.66	1.16
Campylobacter curvus	16577.18	9672.69	1.66	0.97
Veillonella dispar	16183.53	3330.48	1.62	0.33
Veillonella parvula	13397.55	5890.40	1.34	0.59
Veillonella rodentium	11080.72	4463.79	1.11	0.45
Fusobacterium pseudoperiodonticum	10494.90	3323.46	1.05	0.33
Staphylococcus schleiferi	9423.84	3660.56	0.94	0.37
Tannerella forsythia	9143.42	17151.17	0.91	1.72
Porphyromonas gingivalis	7551.02	9371.10	0.76	0.94
Streptococcus sp. LPB0220	7251.15	3565.00	0.73	0.36
Streptococcus gordonii	5660.04	2074.54	0.57	0.21

microorganisms MDPI

c) SMAMP

Species	CPM Mean	CPM STD	% Mean	% STD
Streptococcus salivarius	153220.67	14378.33	15.32	1.44
Fusobacterium nucleatum	146497.13	41387.27	14.65	4.14
Tannerella sp. oral taxon HOT-286	87934.60	18984.27	8.79	1.90
Campylobacter ureolyticus	72213.67	27307.05	7.22	2.73
Megasphaera stantonii	59699.90	10939.55	5.97	1.09
Megasphaera elsdenii	57224.07	8603.13	5.72	0.86
Campylobacter curvus	48111.84	18519.94	4.81	1.85
Neisseria mucosa	30299.36	16366.28	3.03	1.64
Fusobacterium pseudoperiodonticum	29892.26	6090.08	2.99	0.61
Campylobacter gracilis	24643.84	15594.11	2.46	1.56
Streptococcus parasanguinis	18325.99	3738.29	1.83	0.37
Veillonella dispar	14443.35	5594.66	1.44	0.56
Prevotella dentalis	12338.09	4972.50	1.23	0.50
Streptococcus thermophilus	12155.04	1759.81	1.22	0.18
Neisseria subflava	10048.90	9011.30	1.00	0.90
Veillonella parvula	8764.01	1937.77	0.88	0.19
Staphylococcus schleiferi	8143.21	2176.82	0.81	0.22
Veillonella rodentium	7185.88	1666.84	0.72	0.17
Erysipelothrix larvae	6683.64	2938.01	0.67	0.29
Lachnoanaerobaculum umeaense	6485.54	2023.36	0.65	0.20

Table S6: About 950 000 to 1 500 000 sequencing reads passing the ONT Q7 quality score were obtained for each sample, and of these >95% in each sample mapped to bacterial sequences as per the centrifuge results

Name	Number of raw reads	Classified reads	Chordate reads	Artificial reads	Unclassified reads	Microbial reads	Bacterial reads	Viral reads	Fungal reads	Protozoan reads
CHX	1,472,476	96.7%	0.11%	0%	3.26%	96.6%	96.6%	0.000407%	0%	0%
NegCtrl_1	1,294,857	97.2%	0.0306%	0%	2.78%	97.2%	97.1%	0.000309%	0%	0%
NegCtrl_2	1,286,644	97.9%	0.0113%	0%	2.11%	97.9%	97.9%	0.00109%	0%	0%
NegCtrl_3	1,241,613	97.7%	0.0166%	0%	2.33%	97.7%	97.6%	0.000161%	0%	0%
NEgCtrl_4	1,174,811	98.4%	0.0109%	0%	1.64%	98.3%	98.3%	0%	0%	0%
PSB_1	1,185,297	97.7%	0.0136%	0%	2.28%	97.7%	97.7%	0.000253%	0%	0%
PSB_2	1,271,530	98.4%	0.011%	0%	1.65%	98.3%	98.3%	0.000315%	0%	0%
PSB_3	1,342,889	97.6%	0.0157%	0%	2.37%	97.6%	97.6%	0.00119%	0%	0%
PSB_4	1,001,763	98.2%	0.0197%	0%	1.77%	98.2%	98.2%	0.0002%	0%	0%
PZI_1	1,033,474	97.7%	0.0113%	0%	2.34%	97.6%	97.6%	0.0000968%	0%	0%
PZI_2	1,046,485	97.9%	0.0118%	0%	2.07%	97.9%	97.9%	0.000191%	0%	0%
PZI_3	1,122,866	98.6%	0.00989%	0%	1.39%	98.6%	98.6%	0.000267%	0%	0%
PZI_5	1,000,959	98.4%	0.0126%	0%	1.63%	98.4%	98.4%	0.000699%	0%	0%
SMAMP_1	1,007,157	98.6%	0.0109%	0%	1.38%	98.6%	98.6%	0%	0%	0%
SMAMP_2	1,162,149	98.4%	0.00955%	0%	1.64%	98.3%	98.3%	0.000602%	0%	0%
SMAMP_3	1,352,278	98%	0.011%	0%	2.01%	98%	98%	0.000148%	0%	0%
SMAMP_4	950,395	98.3%	0.00705%	0%	1.66%	98.3%	98.3%	0.000316%	0%	0%
SMAMP_5	1,302,290	98.7%	0.00829%	0%	1.34%	98.7%	98.6%	0.000384%	0%	0%

microorganisms

MDPI

Figure S1: Z-section galleries of representative confocal laser scanning microscopic (CLSM) images depicting the ex vivo biofilm formation (3 days) after live/dead staining. The panels illustrate the live (green) and dead (red) microbial populations of biofilms grown on the PSB coating. The multiple Z-sections in the panels were generated by vertical sectioning at 2.0-µm intervals through the sample above the PSB surface. Bars, 20 µm."

PSB

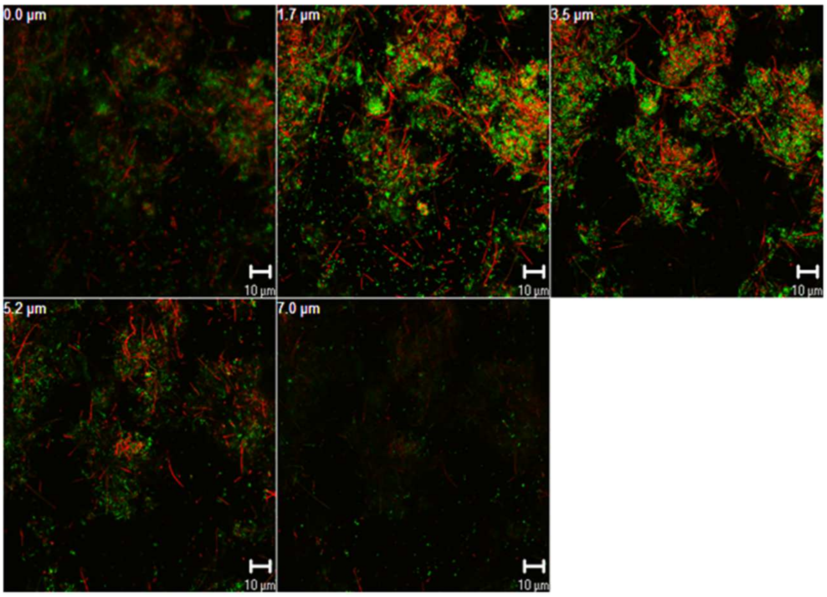

microorganisms

Figure S2: Alpha (A) and beta (B) diversity measures were calculated using the R package vegan (Community Ecology Package, R package version 2.5-7., https://CRAN.R-project.org/package=vegan, published by J. Oksanen, F. Guillaume Blanchet, Michael Friendly, Roeland Kindt, Pierre Legendre, Dan McGlinn, Peter R. Minchin, R. B. O'Hara, Gavin L. Simpson, Peter Solymos, M. Henry H. Stevens, Eduard Szoecs and Helene Wagner 2020) and visualized using the ggplot and pheatmap packages (Raivo Kolde 2019: R package version 1.0.12. https://CRAN.R-project.org/package=pheatmap)

A

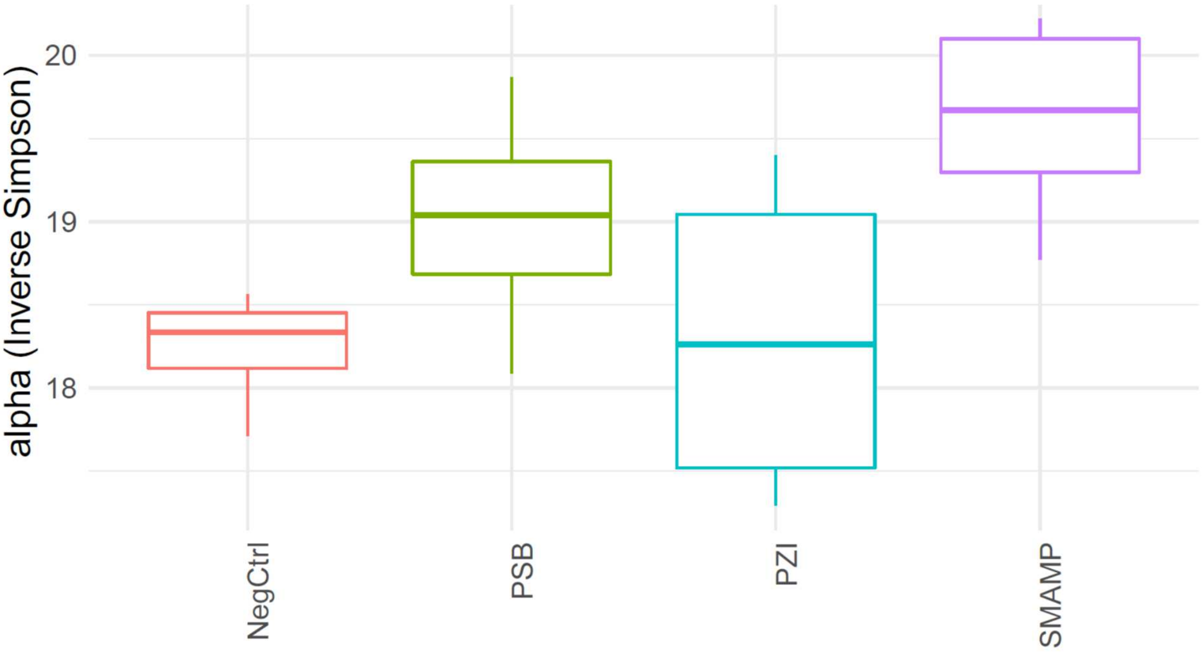

B

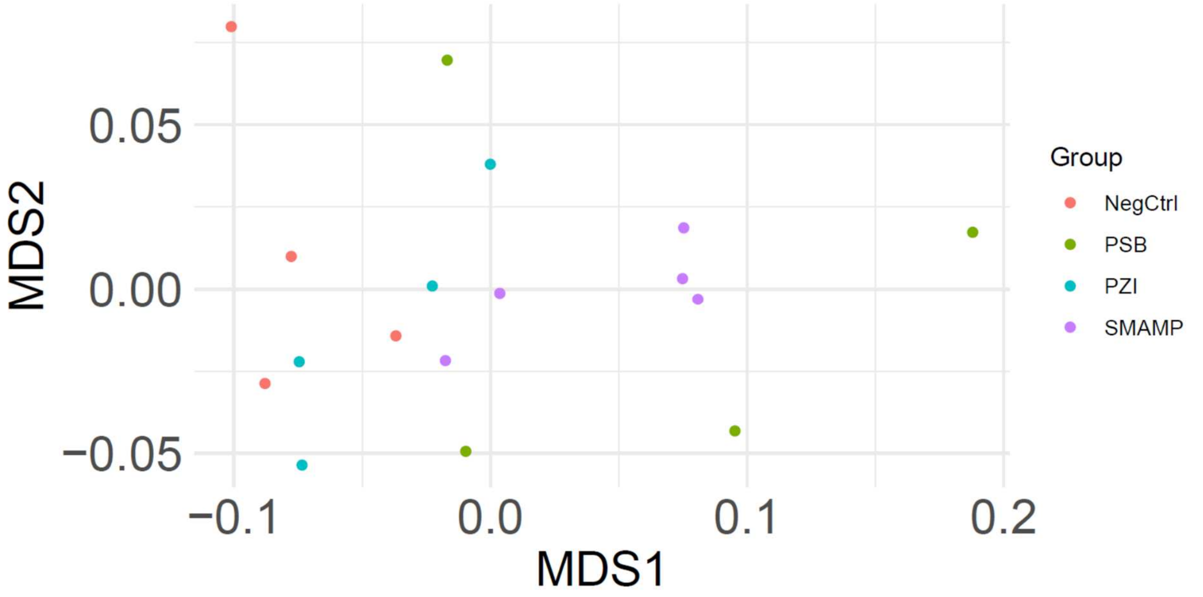

Supplement: Supplementary file 1 [file microorganisms-09-01427-s001.zip › microorganisms-1188596-supplementary.pdf]
